# Identifying the factors promoting colorectal cancer screening uptake in Hong Kong using Andersen’s behavioural model of health services use

**DOI:** 10.1186/s12889-022-13634-7

**Published:** 2022-06-20

**Authors:** Dorothy N. S. Chan, K. C. Choi, Doreen W. H. Au, Winnie K. W. So

**Affiliations:** The Nethersole School of Nursing, 7/F, Esther Lee Building, the Chinese University of Hong Kong, Hong Kong SAR, China

**Keywords:** Colorectal cancer, Screening, Faecal occult blood test, Colonoscopy

## Abstract

**Background:**

Colorectal cancer (CRC) screening is an effective strategy to aid early cancer detection. However, the decision to undergo screening can be affected by a variety of factors. The aims of this study were to examine current CRC screening uptake in Hong Kong and identify the factors associated with it using Andersen’s Behavioural Model as a guiding framework.

**Methods:**

This cross-sectional study was conducted in Hong Kong from August 2019 to December 2020. A sample of 1317 Chinese individuals aged 50 to 75 years were recruited and completed a survey to identify predisposing, enabling, and need-for-care factors, and the colorectal cancer screening uptake rate (faecal occult blood test [FOBT] or faecal immunochemical test [FIT] and colonoscopy) was determined.

**Results:**

The FOBT/FIT uptake rate was 43.9%, while that of the colonoscopy was 26.0%. The provision of a government subsidy for screening and the provision of information booklets were the most significant and second most significant enabling factors for FOBT/FIT uptake, respectively. Visiting a doctor five times or more in the previous year and being recommended to undergo a CRC screening by a doctor, were the most significant enabling factors for colonoscopy uptake. Age, the perceived benefit of and barriers to screening were important predisposing factors for FOBT/FIT and colonoscopy uptake.

**Conclusions:**

Screening uptake rates in Hong Kong have significantly increased over the last decade, although they remain lower than those in other countries. Continual efforts are warranted to promote government-subsidised screening. Relevant educational materials that address the barriers identified in this study should be developed and disseminated to the public.

## Introduction

Over 1.9 million new colorectal cancer (CRC) cases and 935,000 CRC-related deaths were reported worldwide in 2020, the latter accounting for ~ 10% of all cancer-associated deaths [1]. Despite being the third most common cancer globally, CRC is the most common cancer in men and the second most common cancer in women in Hong Kong. According to the most recent data, 5556 new CRC cases were diagnosed in Hong Kong in 2019, accounting for 15.8% of the total new cancer cases, which is slightly higher than the global rate [2]. Accounting for the fact that CRC incidence and mortality increases with age, the age-standardised incidence rates for males and females in 2019 were 42.1 and 25.6 per 100,000, respectively [2].

CRC is often asymptomatic in its early stages, meaning preventive measures such as screening play an important role in detecting and preventing the disease. The most popular CRC screening methods are the faecal occult blood test (FOBT), faecal immunochemical test (FIT) (an improved version of FOBT), sigmoidoscopy, and colonoscopy. According to the Hong Kong Cancer Expert Working Group, average-risk individuals aged 50 to 75 years should seek medical advice and consider annual or biennial FOBT screening, sigmoidoscopies every 5 years, or colonoscopies every 10 years [3]. A Cochrane review found that FOBT screening could reduce CRC mortality by 16% in average-risk individuals, while colonoscopies were linked to a 61% reduction in CRC mortality [4, 5]. Thus, CRC screening is an important and cost-effective secondary prevention and disease control strategy that can significantly increase survival due to early detection and specific diagnosis.

In Hong Kong, a Colorectal Cancer Screening Programme (CRCSP) was trialled in 2016, regularised in 2018, and was fully implemented in 2020. The programme adopts FIT (an improved version of FOBT) as the primary screening test. The programme seeks to subsidise FIT in the private sector for average-risk Hong Kong citizens born from 1946 to 1955, and in 2018, it was expanded to include younger individuals aged 50 to 75 years for CRC screening every 2 years [6]. Under the CRCSP, participants must select a primary care doctor from the list on the CRCSP website and schedule a consultation appointment. Participants meet the primary care doctor in the private health clinic for their first consultation, during which they are assessed for eligibility and sign a consent form. The doctor then instructs each participant on the purpose of screening and provides information about the primary screening test, FIT. In addition, the participants are informed that they will need to return for a second consultation if the FIT returns a positive result, and of the necessity for a colonoscopy referral. Finally, each participant receives a FIT kit for collecting stool specimens at home [6]. The government covers the first and second consultations and the FIT kit under the CRCSP. Those who need a follow-up colonoscopy because of a positive FIT (≥ 100 ng/mL) [6, 7] do not have to pay because the government offers a ‘Standard Package of Colonoscopy Service’ that includes one pre-procedural consultation, one colonoscopy examination and one post-procedural consultation. When colonoscopy specialists are required to provide care or management of complications not covered by the standard package, they may charge a co-payment of up to HK$1000 (~US$133). The primary care doctors and colonoscopy specialists can submit payment claims to the government on a monthly basis for consultations and services provided in the previous month [6].

Since the launch of the CRCSP, the government has made several efforts to promote the screening programme through educational and promotional videos, printed materials (e.g., pamphlets and posters) and television advertisements. A total of 8724 out of 66,697 participants (~ 13%) had a positive FIT in the pilot programme, with 7203 of them going on to have a colonoscopy. Adenoma was detected in 68.9% of these individuals, while adenocarcinoma was subsequently diagnosed in 6.4% of them [8]. Despite recent improvements in the detection of adenoma and adenocarcinoma, the CRC screening uptake rate was only 8.3% in the Hong Kong pilot programme. In 2018/19, the Department of Health (DH) conducted the Health Behaviour Survey to collect information on major health-related behaviours associated with the prevention of non-communicable diseases. This survey revealed that the FOBT/FIT and colonoscopy uptake rates among individuals aged 50 to 75 years were 18.6% (female: 18.3%; male: 19.0%) and 18.1% (female: 18.2%; male: 18.1%), respectively [9]. There figures are still lower than in other Asian countries, where uptake ranged from 21.0 to 62.9% [10].

The decision to undergo screening tests can be influenced by a variety of factors. Leung et al. [11] conducted a review to identify factors contributing to CRC screening uptake among Chinese people living in Western countries, Hong Kong, and other Asian countries. In this review, factors such as knowledge, risk perception, presence of regular primary care providers, doctor recommendations, influence from family and friends, and having undergone other screening tests positively associated with screening uptake [11]. In addition to sociodemographic and psychosocial factors, our earlier study revealed that CRC screening uptake was linked to perceived health status, smoking status, regular medical visits, and the use of alternative medicines [12]. However, as the data used in this previous study were collected over a decade ago in Hong Kong, more recent data are required to account for the changing healthcare climate, in particular the provision of subsidised CRCSP, and guide future screening promotion and improvements.

Given that a variety of factors influence CRC screening, a multifactorial theoretical model could aid the development of strategies to increase screening uptake among average-risk individuals. Therefore, this research used Andersen’s Behavioural Model of Health Services Use (hereafter, Andersen’s Model), a well-established model that considers both individual and contextual variables in the use of healthcare services. This model includes predisposing, enabling and need-for-care factors as major conceptual components and explaining how these factors impact affect the use of health services. Furthermore, the model highlights the importance of measures of access to care or services (potential or realised access, equitable or inequitable access). Potential access refers to the presence of enabling resources such as health insurance and regular sources of care. Realised access refers to the actual use of health services. Equitable access occurs when socio-demographic characteristics and need-for-care account for most health service usage; in contrast, inequitable access occurs when the social structure, health-related beliefs and the presence or absence of enabling resources determine who can obtain health services [13]. The model has been used extensively in many cancer screening studies to examine the relationships among predisposing (demographic factors, social structure, and health-related beliefs), enabling (individual and community resources supporting a person’s ability to access healthcare services), and need-for-care (one’s perceived and assessed health status) factors, in addition to their effects on healthcare service usage and cancer screening [14–17]. Factors found to be significantly associated with CRC screening could be used to guide future CRC screening interventions and counselling. Thus, the aims of this study were to examine the current CRC screening uptake in Hong Kong and identify factors associated with it using Andersen’s Model as a guiding framework.

## Method

### Study design

This was a cross-sectional study conducted from August 2019 to December 2020.

### Study participants and setting

To be included in the study, the participants had to meet the following inclusion criteria: (1) Hong Kong Chinese individuals aged 50 to 75 years; (2) no symptoms suggestive of colorectal cancer, such as a change in bowel habits in the past month, melena, or weight loss of more than 5 kg in the past 6 months; (3) no history of colorectal cancer; and (4) able to understand or communicate in Cantonese. Participants from different districts of Hong Kong were recruited in the community setting, i.e. via community centres, health centres, and workplaces, using a convenience sampling approach during office hours or when activities were organised in the centres, which made it easier to approach the target participants.

The sample size (*n* = 1316) was estimated based on a previous survey of FOBT uptake rate (19%) among Chinese individuals aged 50 years and above [9] and according to the guidelines of Peduzzi et al. [18] for sample size requirements of multivariable logistic regressions. This sample size allowed up to 25 candidate-independent variables to be examined simultaneously and was adequate to detect a binary factor with an odds ratio as small as 1.47 and a normally distributed continuous factor with an odds ratio as small as 1.20 with 80% power and a one-sided significance level of 0.25.

### Study measures

We developed a structured survey containing 56 questions, which used Andersen’s Behavioral Model to address predisposing, enabling, and need-for-care factors and healthcare service utilisation. Predisposing factors included age, sex, marital status, educational level, employment status, and health-related beliefs. Health-related beliefs were related to perceived risk of CRC, perceived severity of the disease, perceived benefit of screening, and perceived psychological and knowledge barriers to screening. Perceived risk was assessed using a statement of whether they were at risk of contracting CRC, which was scored on a 5-point Likert scale from 1 (strongly disagree) to 5 (strongly agree). Perceived severity and fear were assessed using six items scored on a 5-point Likert scale, with higher scores indicating higher perceived severity or fear of the disease. The items showed good internal consistency (Cronbach’s alpha = 0.86) [19]. Perceived benefit of screening was assessed using a statement of whether screening could identify CRC early, while perceived psychological and knowledge barriers to screening were assessed using 13 items. In both cases, the items were scored on a 5-point Likert scale from 1 (strongly disagree) to 5 (strongly agree). The items showed good internal consistency (Cronbach’s alpha = 0.74–0.82) [13].

Enabling factors included income, health insurance, presence of a regular primary care service provider, the use of a subsidy from CRCSP, prompting from healthcare providers (doctors and nurses), influence of family and friends, acquisition of CRC screening-related information, and personal health practices. Given the implementation of the subsidised CRCSP in Hong Kong and the provision of multimedia health-related materials, CRC screening-related information acquisition was assessed using five questions relating to whether the participants had received information from community centres, media (television, newspaper, or social media), booklets, promotional videos, or health talks. Additionally, the utilisation of the CRCSP subsidy to undergo CRC screening was assessed. Based on a literature review [11] of personal health practices influencing CRC screening uptake among the Chinese, personal health practices were assessed using four questions relating to whether they had a primary care service provider, and, if so, the accessibility of CRC screening at their primary care service providers, whether they had undergone screening for other cancers, and the number of doctor’s visits carried out in the past year. Additionally, the use of alternative medicine was also assessed.

The need-for-care factors included a family history of CRC (first- and second-degree relatives diagnosed with CRC) and perceived and evaluated health status. Perceived and evaluated health statuses were assessed using three questions relating to how they would describe their current health (excellent/very good/good/fair/poor), the number of chronic illnesses they currently have, and their smoking status. CRC screening uptake was assessed using two questions asking whether they had ever previously undergone an FOBT/FIT or colonoscopy.

### Data collection procedure

Ethical approval was sought from the ethics committee of the study institution. All experimental protocols were approved by the Joint Chinese University of Hong Kong-New Territories East Cluster Clinical Research Ethics Committee. Potential participants were recruited in the community setting. We contacted the individuals in charge of various community centres, health centres, and workplaces to gain access to potential participants. More than 25 of these community and health centres and workplaces across 14 of the 18 districts in Hong Kong allowed access to potential participants and assisted in recruitment [20]. Our data collectors approached the potential participants, assessed their eligibility, and explained the study purpose. An information sheet was distributed, and written informed consent was obtained when the potential participants agreed to join the study. The Chinese version of the paper-based survey was completed via face-to-face interviews conducted by the data collectors. During the study period, data collection was affected by the COVID-19 pandemic and some of the collaborating centres were closed. Thus, the telephone numbers of the participants were obtained, and the participants were contacted by our data collectors. After assessment of the participants’ eligibility and obtaining their verbal consent (This consent procedure was approved by the ethics committee.), around 717 surveys were completed via telephone interviews conducted by the data collectors, who documented the responses using the paper-based survey. Each survey was completed in approximately 15 to 20 minutes.

### Statistical analysis

Data were summarised and presented using appropriate descriptive statistics and analysed using IBM SPSS 25.0 (IBM Crop., Armonk, NY). The primary outcome variables of the study were CRC screening uptake, namely FOBT/FIT and colonoscopy. Logistic regression was used to examine influencing factors associated with each outcome. Univariate analyses were carried out for each of the potential influencing factors associated with each outcome using binary logistic regressions. Factors with *p* < 0.25 were selected as candidate independent variables, and multivariable logistic regressions were used to identify factors independently associated with each outcome. The goodness-of-fit of the multivariable logistic regression models was assessed using the Hosmer-Lemeshow test [21], and the results of each model were presented by the odds ratios (OR) and their associated 95% confidence intervals (CI) of the factors retained in the model. All statistical tests were two-sided with a significance level of 0.05.

## Results

### Recruitment and response rate

A total of 1948 eligible Chinese individuals aged 50 to 75 years were approached during the study period. Of them, 610 refused to participate, and 21 withdrew from the study. In total, 1317 participants completed the survey. The response rate was 67.6%.

### Participants’ characteristics

The average age of the participants was 64.8 (SD = 7.1) years. Over half of the participants were female (59.7%), married or previously married (92.2%), had a secondary or lower education level (81.5%), and had no part-time or full-time employment (70.7%). Additionally, over half of the participants (55.2%) made a monthly income of less than HK$10,000 (~US$1316). Most participants had no health insurance (68.6%). More than half of the participants had a regular primary care service provider (65.8%). Nearly 87% of the participants did not have a family history of CRC. Approximately 52% of the participants considered their health to be good or very good, and around 81% of them had never smoked (Tables 1, 2, 3).

**Table 1:** Characteristics of the predisposing factors of the Chinese aged 50 to 75 who were eligible for colorectal cancer screening (*N* = 1317)

	Mean (SD) / n (%)
Predisposing factors	
Age (years)^†^	64.8 (7.1)
Sex	
Male	531 (40.3%)
Female	786 (59.7%)
Marital status	
Never married	102 (7.7%)
Previously married	120 (9.1%)
Married	1095 (83.1%)
Educational level	
No formal education / primary	475 (36.1%)
Secondary	598 (45.4%)
Post-secondary	129 (9.8%)
University	115 (8.7%)
Have a part-time/full-time job	
No	931 (70.7%)
Yes	386 (29.3%)
*Health-related beliefs*	
Perceived risk^†^ [possible range: 1–5]	2.40 (0.82)
Perceived severity^†^ [possible range: 1–5]	2.41 (0.66)
Perceived benefits^†^ [possible range: 1–5]	3.94 (0.84)
Perceived barriers^†^ [possible range: 1–5]	2.47 (0.55)

Data marked with ^†^ are presented as mean (standard deviation), all others are presented as frequency (%)

**Table 2:** Characteristics of the enabling factors of the Chinese aged 50 to 75 who were eligible for colorectal cancer screening (*N* = 1317)

	Mean (SD) / n (%)
Enabling factors	
Monthly household income (HK$)	
< 10,000	727 (55.2%)
10,000 – 19,999	190 (14.4%)
20,000 – 29,999	137 (10.4%)
≥ 30,000	263 (20.0%)
Have health insurance	
No	904 (68.6%)
Yes	413 (31.4%)
*Health practice*	
Have regular primary care service provider	
No	450 (34.2%)
Yes	867 (65.8%)
CRC cancer screening accessible at your primary care service provider	
No	928 (70.5%)
Yes	389 (29.5%)
Number of times visiting a doctor in the past year	
0	250 (19.0%)
1–2	330 (25.1%)
3–4	354 (26.9%)
≥ 5	383 (29.1%)
Had ever undergone other cancer screenings except CRC	
No	908 (68.9%)
Yes	409 (31.1%)
*Utilization of complementary therapies*	
Use of acupuncture	
Not at all/ a little	1063 (80.7%)
Sometimes	174 (13.2%)
Often/ always	80 (6.1%)
Use of cupping	
Not at all/ a little	1152 (87.5%)
Sometimes	123 (9.3%)
Often/ always	42 (3.2%)
Use of Chinese herbal medicine	
Not at all/ a little	886 (67.3%)
Sometimes	320 (24.3%)
Often/ always	111 (8.4%)
Use of bonesetting	
Not at all/ a little	1157 (87.9%)
Sometimes	133 (10.1%)
Often/ always	27 (2.1%)
Use of tuina (Chinese massage)	
Not at all/ a little	983 (74.6%)
Sometimes	232 (17.6%)
Often/ always	102 (7.7%)
*CRC screening-related information acquisition:*	
Do you know there is government subsidy from CRC screening programme	
No	267 (20.3%)
Yes	1050 (79.7%)
Had ever used the government subsidy for CRC screening	
No	885 (67.2%)
Yes	432 (32.8%)
Had ever received information about cancer prevention and screening from community centres	
No	1045 (79.3%)
Yes	272 (20.7%)
Had ever received information about cancer prevention and screening from media	
No	353 (26.8%)
Yes	964 (73.2%)
Had ever received information about cancer prevention and screening from booklets	
No	955 (72.5%)
Yes	362 (27.5%)
Had ever received information about cancer prevention and screening from promotion video	
No	551 (41.8%)
Yes	766 (58.2%)
Had ever received information about cancer prevention and screening from health talks	
No	1038 (78.8%)
Yes	279 (21.2%)
*Prompt to have a CRC screening*	
Had ever prompted by doctor to have a CRC screening	
No	767 (58.2%)
Yes	550 (41.8%)
Had ever prompted by nurse to have a CRC screening	
No	1086 (82.5%)
Yes	231 (17.5%)
Had ever prompted by friend to have a CRC screening	
No	1029 (78.1%)
Yes	288 (21.9%)
Had ever prompted by family to have a CRC screening	
No	923 (70.1%)
Yes	394 (29.9%)

Data are presented as frequency (%)

**Table 3:** Characteristics of the needs factors of the Chinese aged 50 to 75 who were eligible for colorectal cancer screening (*N* = 1317)

	n (%)
Needs Factors	
Family history of colorectal cancer	
No	1145 (86.9%)
Yes	172 (13.1%)
*Health status*	
Perceived health status	
Excellent/very good	148 (11.2%)
Good	535 (40.6%)
Fair	562 (42.7%)
Poor	72 (5.5%)
Number of chronic diseases	
None	623 (47.3%)
1	384 (29.2%)
2	166 (12.6%)
≥ 3	144 (10.9%)
Smoking status	
Never smoke	1061 (80.6%)
Ex-smoker	156 (11.8%)
Current smoker	100 (7.6%)

Data are presented as frequency (%)

Approximately 73% of the participants received CRC screening-related information from the media. Although nearly 80% of them claimed to know about the government subsidies through the CRCSP, only 32.8% of them reported to have used the subsidies. Around 42% of them had been prompted by their doctors to undergo CRC screening (Table 2).

### CRC screening uptake

Overall, 43.9% of the participants had previously undergone an FOBT/FIT (female: 47.7%; male: 38.2%), while 26.0% of them had previously undergone a colonoscopy to screen for CRC (female: 25.1%; male: 27.3%) (Tables 4, 5, 6).

**Table 4:** CRC screening participation

	n	Prevalence (95% CI)
Ever had a Fecal Occult blood test/ Fecal Immunochemical Test	578	43.9% (41.2–46.6%)
Ever had a colonoscopy	342	26.0% (23.6–28.4%)

*CI* confidence interval

**Table 5:** Factors associated with ever had a Fecal Occult Blood Test (FOBT)/ Fecal Immunochemical Test (FIT)

	Ever had a FOBT/FIT					
	No (*n* = 739)	Yes (*n* = 578)	OR_U_	*p*-value	OR_A_ (95% CI)	*p*-value
***Predisposing factors***						
Age (years)^†^	63.8 (7.7)	66.1 (6.0)	1.61	< 0.001	1.34 (1.07–1.67)	0.010
Sex						
Male (ref)	328 (61.8%)	203 (38.2%)	1		NR	
Female	411 (52.3%)	375 (47.7%)	1.47	0.001		
Marital status						
Never married (ref)	53 (52.0%)	49 (48.0%)	1		NR	
Previously married	77 (64.2%)	43 (35.8%)	0.60	0.067		
Married	609 (55.6%)	486 (44.4%)	0.86	0.478		
Educational level						
No formal education / primary (ref)	266 (56.0%)	209 (44.0%)	1		NE	
Secondary	341 (57.0%)	257 (43.0%)	0.96	0.737		
Post-secondary	71 (55.0%)	58 (45.0%)	1.04	0.845		
University	61 (53.0%)	54 (47.0%)	1.13	0.567		
Have a part-time/full-time job						
No (ref)	476 (51.1%)	455 (48.9%)	1		NR	
Yes	263 (68.1%)	123 (31.9%)	0.49	< 0.001		
Health-related beliefs						
Perceived risk^†^	2.37 (0.80)	2.44 (0.84)	1.10	0.168	NR	
Perceived severity^†^	2.37 (0.69)	2.47 (0.63)	1.25	0.009	1.50 (1.15–1.96)	0.003
Perceived benefits^†^	3.79 (0.85)	4.13 (0.80)	1.69	< 0.001	1.44 (1.19–1.74)	< 0.001
Perceived barriers^†^	2.57 (0.55)	2.34 (0.52)	0.46	< 0.001	0.40 (0.29–0.54)	< 0.001
***Enabling factors***						
Monthly household income (HK$)						
< 10,000 (ref)	374 (51.4%)	353 (48.6%)	1		NR	
10,000 – 19,999	114 (60.0%)	76 (40.0%)	0.71	0.036		
20,000 – 29,999	93 (67.9%)	44 (32.1%)	0.50	< 0.001		
≥ 30,000	158 (60.1%)	105 (39.9%)	0.70	0.016		
Have health insurance						
No (ref)	515 (57.0%)	389 (43.0%)	1		NE	
Yes	224 (54.2%)	189 (45.8%)	1.12	0.354		
Health practice						
Have regular primary care service provider						
No (ref)	301 (66.9%)	149 (33.1%)	1		NR	
Yes	438 (50.5%)	429 (49.5%)	1.98	< 0.001		
CRC cancer screening accessible at your primary care service provider						
No (ref)	597 (64.3%)	331 (35.7%)	1		NR	
Yes	142 (36.5%)	247 (63.5%)	3.14	< 0.001		
Frequency of visiting a doctor in the past year						
0 (ref)	182 (72.8%)	68 (27.2%)	1		NR	
1–2	184 (55.8%)	146 (44.2%)	2.12	< 0.001		
3–4	189 (53.4%)	165 (46.6%)	2.34	< 0.001		
≥ 5	184 (48.0%)	199 (52.0%)	2.90	< 0.001		
Had ever undergone other cancer screenings except CRC						
No (ref)	554 (61.0%)	354 (39.0%)	1		1	
Yes	185 (45.2%)	224 (54.8%)	1.90	< 0.001	1.97 (1.42–2.73)	< 0.001
Utilization of complementary therapies						
Use of acupuncture						
Not at all/ a little (ref)	609 (57.3%)	454 (42.7%)	1		NR	
Sometimes	89 (51.1%)	85 (48.9%)	1.28	0.131		
Often/ always	41 (51.2%)	39 (48.8%)	1.28	0.294		
Use of cupping						
Not at all/ a little (ref)	645 (56.0%)	507 (44.0%)	1		NE	
Sometimes	73 (59.3%)	50 (40.7%)	0.87	0.475		
Often/ always	21 (50.0%)	21 (50.0%)	1.27	0.444		
Use of Chinese herbal medicine						
Not at all/ a little (ref)	509 (57.4%)	377 (42.6%)	1		NE	
Sometimes	170 (53.1%)	150 (46.9%)	1.19	0.182		
Often/ always	60 (54.1%)	51 (45.9%)	1.15	0.496		
Use of bonesetting						
Not at all/ a little (ref)	638 (55.1%)	519 (44.9%)	1		NR	
Sometimes	82 (61.7%)	51 (38.3%)	0.77	0.153		
Often/ always	19 (70.4%)	8 (29.6%)	0.52	0.122		
Use of tuina (Chinese massage)						
Not at all/ a little (ref)	525 (53.4%)	458 (46.6%)	1		NR	
Sometimes	148 (63.8%)	84 (36.2%)	0.65	0.004		
Often/ always	66 (64.7%)	36 (35.3%)	0.63	0.030		
CRC screening-related information acquisition:						
Do you know there is government subsidy from CRC screening programme						
No (ref)	198 (74.2%)	69 (25.8%)	1		NR	
Yes	541 (51.5%)	509 (48.5%)	2.70	< 0.001		
Had ever used the government subsidy for CRC screening						
No (ref)	691 (78.1%)	194 (21.9%)	1		1	
Yes	48 (11.1%)	384 (88.9%)	28.50	< 0.001	23.87 (16.48–34.56)	< 0.001
Had ever received information about cancer prevention and screening from community centres						
No (ref)	637 (61.0%)	408 (39.0%)	1		NR	
Yes	102 (37.5%)	170 (62.5%)	2.60	< 0.001		
Had ever received information about cancer prevention and screening from media						
No (ref)	233 (66.0%)	120 (34.0%)	1		NR	
Yes	506 (52.5%)	458 (47.5%)	1.76	< 0.001		
Had ever received information about cancer prevention and screening from booklets						
No (ref)	626 (65.5%)	329 (34.5%)	1		1	
Yes	113 (31.2%)	249 (68.8%)	4.19	< 0.001	2.22 (1.56–3.16)	< 0.001
Had ever received information about cancer prevention and screening from promotion video						
No (ref)	359 (65.2%)	192 (34.8%)	1		NR	
Yes	380 (49.6%)	386 (50.4%)	1.90	< 0.001		
Had ever received information about cancer prevention and screening from health talks						
No (ref)	651 (62.7%)	387 (37.3%)	1		NR	
Yes	88 (31.5%)	191 (68.5%)	3.65	< 0.001		
Prompt to have a CRC screening						
Had ever prompted by doctor to have a CRC screening						
No (ref)	450 (58.7%)	317 (41.3%)	1		1	
Yes	289 (52.5%)	261 (47.5%)	1.28	0.027	1.46 (1.04–2.05)	0.029
Had ever prompted by nurse to have a CRC screening						
No (ref)	646 (59.5%)	440 (40.5%)	1		NR	
Yes	93 (40.3%)	138 (59.7%)	2.18	< 0.001		
Had ever prompted by friend to have a CRC screening						
No (ref)	619 (60.2%)	410 (39.8%)	1		NR	
Yes	120 (41.7%)	168 (58.3%)	2.11	< 0.001		
Had ever prompted by family to have a CRC screening						
No (ref)	551 (59.7%)	372 (40.3%)	1		1	
Yes	188 (47.7%)	206 (52.3%)	1.62	< 0.001	1.70 (1.19–2.42)	0.004
***Needs factors***						
Family history of colorectal cancer						
No (ref)	650 (56.8%)	495 (43.2%)	1		NR	
Yes	89 (51.7%)	83 (48.3%)	1.23	0.216		
Health status						
Perceived health status						
Excellent/very good (ref)	73 (49.3%)	75 (50.7%)	1		NR	
Good	313 (58.5%)	222 (41.5%)	0.69	0.047		
Fair	322 (57.3%)	240 (42.7%)	0.73	0.083		
Poor	31 (43.1%)	41 (56.9%)	1.29	0.383		
Number of chronic diseases						
None (ref)	365 (58.6%)	258 (41.4%)	1		NE	
1	211 (54.9%)	173 (45.1%)	1.16	0.257		
2	85 (51.2%)	81 (48.8%)	1.35	0.088		
≥ 3	78 (54.2%)	66 (45.8%)	1.20	0.333		
Smoking status						
Never smoke (ref)	554 (52.2%)	507 (47.8%)	1		1	
Ex-smoker	101 (64.7%)	55 (35.3%)	0.60	0.004	0.76 (0.46–1.26)	0.291
Current smoker	84 (84.0%)	16 (16.0%)	0.21	< 0.001	0.29 (0.14–0.61)	0.001

Data marked with ^†^ are presented as mean (standard deviation), all others are presented as frequency (row %)

*ref* reference group of the categorical variable, OR_U_ univariate odds ratio, OR_A_ odds ratio adjusted for other significant factors obtained from backward multivariable logistic regression analysis using variables with *p*-value < 0.25 in univariate analysis as candidate variables, *NE* not entered into multivariable analysis, *NR* not retained in backward multivariable logistic regression

Odds ratio for age was estimated per 10-year increment

**Table 6:** Factors associated with ever had a colonoscopy

	Ever had a colonoscopy					
	No (*n* = 975)	Yes (*n* = 342)	OR_U_	p-value	OR_A_ (95% CI)	*p*-value
***Predisposing factors***						
Age (years)^†^	64.7 (7.1)	65.3 (7.2)	1.13	0.179	1.41 (1.08–1.84)	0.012
Sex						
Male (ref)	386 (72.7%)	145 (27.3%)	1		NE	
Female	589 (74.9%)	197 (25.1%)	0.89	0.363		
Marital status						
Never married (ref)	74 (72.5%)	28 (27.5%)	1		NE	
Previously married	93 (77.5%)	27 (22.5%)	0.77	0.395		
Married	808 (73.8%)	287 (26.2%)	0.94	0.785		
Educational level						
No formal education / primary (ref)	362 (76.2%)	113 (23.8%)	1		NR	
Secondary	451 (75.4%)	147 (24.6%)	1.04	0.763		
Post-secondary	89 (69.0%)	40 (31.0%)	1.44	0.096		
University	73 (63.5%)	42 (36.5%)	1.84	0.006		
Have a part-time/full-time job						
No (ref)	679 (72.9%)	252 (27.1%)	1		NR	
Yes	296 (76.7%)	90 (23.3%)	0.82	0.158		
***Health-related beliefs***						
Perceived risk^†^	2.39 (0.80)	2.43 (0.88)	1.06	0.428	NE	
Perceived severity^†^	2.40 (0.66)	2.44 (0.69)	1.09	0.350	NE	
Perceived benefits^†^	3.87 (0.85)	4.16 (0.78)	1.60	< 0.001	1.40 (1.18–1.66)	< 0.001
Perceived barriers^†^	2.54 (0.53)	2.27 (0.55)	0.40	< 0.001	0.37 (0.29–0.48)	< 0.001
***Enabling factors***						
Monthly household income (HK$)						
< 10,000 (ref)	526 (72.4%)	201 (27.6%)	1		1	
10,000 – 19,999	166 (87.4%)	24 (12.6%)	0.38	< 0.001	0.53 (0.32–0.88)	0.014
20,000 – 29,999	104 (75.9%)	33 (24.1%)	0.83	0.390	1.05 (0.63–1.76)	0.840
≥ 30,000	179 (68.1%)	84 (31.9%)	1.23	0.188	1.36 (0.89–2.09)	0.153
Have health insurance						
No (ref)	703 (77.8%)	201 (22.2%)	1		1	
Yes	272 (65.9%)	141 (34.1%)	1.81	< 0.001	2.05 (1.48–2.84)	< 0.001
Health practice						
Have regular primary care service provider						
No (ref)	377 (83.8%)	73 (16.2%)	1		1	
Yes	598 (69.0%)	269 (31.0%)	2.32	< 0.001	1.60 (1.16–2.21)	0.004
CRC cancer screening accessible at your primary care service provider						
No (ref)	706 (76.1%)	222 (23.9%)	1		NR	
Yes	269 (69.2%)	120 (30.8%)	1.42	0.009		
Frequency of visiting a doctor in the past year						
0 (ref)	211 (84.4%)	39 (15.6%)	1		1	
1–2	253 (76.7%)	77 (23.3%)	1.65	0.022	1.40 (0.87–2.24)	0.165
3–4	266 (75.1%)	88 (24.9%)	1.79	0.006	1.93 (1.19–3.13)	0.008
≥ 5	245 (64.0%)	138 (36.0%)	3.05	< 0.001	3.53 (2.21–5.65)	< 0.001
Had ever undergone other cancer screenings except CRC						
No (ref)	703 (77.4%)	205 (22.6%)	1		NR	
Yes	272 (66.5%)	137 (33.5%)	1.73	< 0.001		
Utilization of complementary therapies:						
Use of acupuncture						
Not at all/ a little (ref)	803 (75.5%)	260 (24.5%)	1		NR	
Sometimes	120 (69.0%)	54 (31.0%)	1.39	0.066		
Often/ always	52 (65.0%)	28 (35.0%)	1.66	0.038		
Use of cupping						
Not at all/ a little (ref)	862 (74.8%)	290 (25.2%)	1		NR	
Sometimes	84 (68.3%)	39 (31.7%)	1.38	0.117		
Often/ always	29 (69.0%)	13 (31.0%)	1.33	0.399		
Use of Chinese herbal medicine						
Not at all/ a little (ref)	659 (74.4%)	227 (25.6%)	1		NE	
Sometimes	239 (74.7%)	81 (25.3%)	0.98	0.914		
Often/ always	77 (69.4%)	34 (30.6%)	1.28	0.259		
Use of bonesetting						
Not at all/ a little (ref)	854 (73.8%)	303 (26.2%)	1		NE	
Sometimes	98 (73.7%)	35 (26.3%)	1.01	0.975		
Often/ always	23 (85.2%)	4 (14.8%)	0.49	0.191		
Use of tuina (Chinese massage)						
Not at all/ a little (ref)	743 (75.6%)	240 (24.4%)	1		NR	
Sometimes	162 (69.8%)	70 (30.2%)	1.34	0.071		
Often/ always	70 (68.6%)	32 (31.4%)	1.42	0.124		
CRC screening-related information acquisition						
Do you know there is government subsidy from CRC screening programme						
No (ref)	209 (78.3%)	58 (21.7%)	1		NR	
Yes	766 (73.0%)	284 (27.0%)	1.34	0.077		
Had ever used the government subsidy for CRC screening						
No (ref)	674 (76.2%)	211 (23.8%)	1		NR	
Yes	301 (69.7%)	131 (30.3%)	1.39	0.012		
Had ever received information about cancer prevention and screening from community centres						
No (ref)	774 (74.1%)	271 (25.9%)	1		NE	
Yes	201 (73.9%)	71 (26.1%)	1.01	0.955		
Had ever received information about cancer prevention and screening from media						
No (ref)	283 (80.2%)	70 (19.8%)	1		NR	
Yes	692 (71.8%)	272 (28.2%)	1.59	0.002		
Had ever received information about cancer prevention and screening from booklets						
No (ref)	727 (76.1%)	228 (23.9%)	1		NR	
Yes	248 (68.5%)	114 (31.5%)	1.47	0.005		
Had ever received information about cancer prevention and screening from promotion video						
No (ref)	431 (78.2%)	120 (21.8%)	1		NR	
Yes	544 (71.0%)	222 (29.0%)	1.47	0.003		
Had ever received information about cancer prevention and screening from health talks						
No (ref)	782 (75.3%)	256 (24.7%)	1		NR	
Yes	193 (69.2%)	86 (30.8%)	1.36	0.038		
Prompt to have a CRC screening						
Had ever prompted by doctor to have a CRC screening						
No (ref)	613 (79.9%)	154 (20.1%)	1		1	
Yes	362 (65.8%)	188 (34.2%)	2.07	< 0.001	2.19 (1.65–2.91)	< 0.001
Had ever prompted by nurse to have a CRC screening						
No (ref)	823 (75.8%)	263 (24.2%)	1		NR	
Yes	152 (65.8%)	79 (34.2%)	1.63	0.002		
Had ever prompted by friend to have a CRC screening						
No (ref)	777 (75.5%)	252 (24.5%)	1		NR	
Yes	198 (68.8%)	90 (31.3%)	1.40	0.021		
Had ever prompted by family to have a CRC screening						
No (ref)	706 (76.5%)	217 (23.5%)	1		NR	
Yes	269 (68.3%)	125 (31.7%)	1.51	0.002		
***Needs factors***						
Family history of colorectal cancer						
No (ref)	866 (75.6%)	279 (24.4%)	1		1	
Yes	109 (63.4%)	63 (36.6%)	1.70	0.001	1.78 (1.22–2.60)	0.003
Health status						
Perceived health status						
Excellent/very good (ref)	116 (78.4%)	32 (21.6%)	1		NE	
Good	399 (74.6%)	136 (25.4%)	1.24	0.343		
Fair	407 (72.4%)	155 (27.6%)	1.38	0.144		
Poor	53 (73.6%)	19 (26.4%)	1.30	0.432		
Number of chronic diseases						
None (ref)	472 (75.8%)	151 (24.2%)	1		NR	
1	287 (74.7%)	97 (25.3%)	1.06	0.714		
2	121 (72.9%)	45 (27.1%)	1.16	0.447		
≥ 3	95 (66.0%)	49 (34.0%)	1.61	0.016		
Smoking status						
Never smoke (ref)	776 (73.1%)	285 (26.9%)	1		NR	
Ex-smoker	113 (72.4%)	43 (27.6%)	1.04	0.853		
Current smoker	86 (86.0%)	14 (14.0%)	0.44	0.006		

Data marked with ^†^ are presented as mean (standard deviation), all others are presented as frequency (row %)

ref reference group of the categorical variable, OR_U_ univariate odds ratio; OR_A_ odds ratio adjusted for other significant factors obtained from backward multivariable logistic regression analysis using variables with *p*-value < 0.25 in univariate analysis as candidate variables, *NE* not entered into multivariable analysis, *NR* not retained in backward multivariable logistic regression

Odds ratio for age was estimated per 10-year increment

### Factors associated with FOBT/FIT uptake

The associations between having undergone an FOBT/FIT to screen for CRC and the identified predisposing, enabling, and need-for-care factors were examined (Table 5). Among the predisposing factors, age and health-related beliefs were found to influence FOBT/FIT uptake for CRC screening. Specifically, older people were more likely to have undergone FOBT/FIT (adjusted odds ratio [AOR]: 1.34, 95% CI: 1.07–1.67 for every 10-year increment in age), and participants with higher perceived severity of the disease and benefit of screening had a significantly higher FOBT/FIT uptake, with AORs ranging from 1.44 to 1.50. Participants with higher perceived barriers to screening were less likely to undergo an FOBT/FIT (AOR: 0.40, 95% CI: 0.29–0.54).

Among the enabling factors, participants who (1) had used the government subsidy, (2) had obtained details about cancer prevention and screening from booklets, (3) had undergone screening for cancers other than CRC, or (4) had been encouraged to be screened for CRC by their doctor or family members were more likely to have undergone an FOBT/FIT, with odds ratios ranging from 1.46 to 23.87 (*p* < 0.05). The existence of a government subsidy for screening was the most significant enabling factor for FOBT/FIT uptake, with participants who used the government subsidy for CRC screening being 23 times more likely to undergo an FOBT/FIT than those who did not (AOR: 23.87, 95% CI: 16.48–34.56). Learning about cancer prevention and screening through booklets was the second most significant enabling factor for FOBT/FIT uptake (AOR: 2.22, 95% CI: 1.56–3.16).

Nevertheless, only one need-for-care factor was significantly associated with FOBT/FIT uptake. Participants who were current smokers (AOR: 0.29, 95% CI: 0.14–0.61) were less likely to have undergone an FOBT/FIT compared with those who had never smoked.

### Factors associated with colonoscopy uptake

The associations between having undergone a colonoscopy to screen for CRC and the identified predisposing, enabling, and need-for-care factors were examined (Table 6). Similar to what was observed for FOBT/FIT uptake, participants of older age and those with a higher perceived benefit of screening were more likely to have undergone a colonoscopy while those with higher perceived barriers had a reduced rate of colonoscopy uptake (*p* < 0.05).

Among the enabling factors, participants who (1) had health insurance coverage, (2) had a regular primary care service provider, (3) had visited a doctor more than twice in the previous year, or (4) had been prompted by their doctor to have a CRC screening were more likely to have undergone a colonoscopy (*p* < 0.05). Visiting a doctor five times or more in the previous year (AOR: 3.53, 95% CI: 2.21–5.65), being recommended to undergo CRC screening by a doctor (AOR: 2.19, 95% CI: 1.65–2.91), and having health insurance (AOR: 2.05, 95% CI: 1.48–2.84) were the most significant enabling factors for colonoscopy uptake. However, participants with a monthly household income of HK$10,000 to HK$19,999 (AOR: 0.53, 95% CI: 0.32–0.88) were significantly less likely to undergo a colonoscopy compared to those who had a monthly household income of less than HK$10,000.

Unlike FOBT/FIT, a family history of CRC was an important need-for-care factor for colonoscopy uptake, with participants having a family history being more likely to have previously undergone a colonoscopy (AOR: 1.78, 95% CI: 1.22–2.60).

## Discussion

This study sought to provide an updated view of how individual and contextual factors are linked to FOBT/FIT and colonoscopy uptake for CRC screening in Hong Kong. Using Andersen’s Model as a guiding framework, our findings contribute to the knowledge surrounding the enabling factors associated with CRC screening. Among the Hong Kong Chinese participants in this study, 43.9% had undergone an FOBT/FIT, and 26.0% had undergone a colonoscopy. There was no significant difference in FOBT/FIT and colonoscopy uptake between men and women. The uptake rate of CRC screening identified in this study is significantly higher than that reported in our previous study conducted more than a decade ago and in the Health Behaviour Survey 2018/19 [9, 12]. Despite this increase, the uptake rate in the Hong Kong population remains lower than that in Koreans (45.7%), aggregated Asian Americans (47.0–58.0%), non-Hispanic white Americans (66.0%), and the general population of the United States (59.0%) [22].

Several enabling factors were discovered in this study that could predict CRC screening uptake. The use of the government screening subsidy was the most significant predictor identified by this study’s analytical model, with participants who used subsidy being 23 times more likely than those who did not to undergo an FOBT/FIT for CRC screening. In Hong Kong, there is no quota for the number of participants under the CRCSP, and CRC screening is heavily subsidised by the government. A government subsidy of HK$280 (~US$37) per consultation is available, including for the second consultation to follow up on a positive FIT test result. Under the standard colonoscopy service package targeted at FIT-positive participants, a subsidy of HK$8500 (~US$1133) is available if polyp removal is necessary, while HK$7800 (~US$1040) is available if no polyp removal is required. Colonoscopy specialists may charge a co-payment not exceeding HK$1000 (~US$133) when, as a result of complications, they must provide care or management not covered by the standard government-subsidised package of colonoscopy services [6].

According to the CRCSP’s most recent data, 649 primary care doctors have been enrolled across 961 clinics since 2016. A co-payment was not required at 97% of these clinics. Similarly, 160 colonoscopy specialists provide colonoscopy examination services at 312 locations, with 81% of these locations charging no co-payment if no polypectomy is required, and 70% charging no co-payment if a polypectomy is performed [6]. Requiring a co-payment, albeit small, could deter many people from undergoing cancer screening. In Japan, for example, fully subsidised testing has been reported to have the potential to significantly increase hepatitis virus screening rates by encouraging hard-to-reach individuals to get tested [23]. Most of the target demographic of our study, aged 50 to 75 years, were retired, implying that they were no longer actively employed. As a result, having financial support from the CRCSP is a key motivator for increasing screening participation. Removing co-payments, if possible, could encourage screening uptake in hard-to-reach and low-income populations.

While free screening is beneficial, it comes at a significant cost to society. Other alternatives for increasing screening uptake were identified in this study. Our findings agree with those of a previous study, which found that individuals who were given enough information about CRC screening and were given financial assistance through their insurance coverage were more likely to undergo screening [24]. By carrying out separate analyses of FOBT/FIT and colonoscopy uptake, we found that booklets providing information about cancer prevention and screening were the second most important factor in increasing FOBT/FIT uptake, while participants who had received a doctor recommendation and had health insurance coverage were more likely to undergo a colonoscopy examination. Most of the target individuals with a lower educational level (primary or below) had less access to information about CRC screening and prevention. It is reasonable to expect that those with a lower educational level will require simpler text. Therefore, increasing the accessibility of the reading material will aid their acquisition of CRC screening-related knowledge. Furthermore, our findings are in line with our other studies, emphasising the importance of doctors continuing to advise their patients about the importance of FIT and colonoscopy screening, particularly for those with a family history of CRC [25]. Clients’ successful completion of screening could be attributed to the doctors’ encouragement, and both the doctors and clients worked together on shared and informed decision making to achieve the goal of screening uptake [26]. Consistent with previous research on access to care, two other important factors that enhanced colonoscopy use were health insurance and a regular primary care provider [27]. These enabled access to health services, which is the prerequisite for realised access to occur. According to DeVoe et al., if these factors synergise, meaning that both financing access and delivery access are consistently available and well-coordinated, this could lead to real access [27].

As reported previously, we found that perceived psychological barriers and knowledge barriers to screening were linked to a lower likelihood of undergoing both the FOBT/FIT and colonoscopy examinations [28–30]. In our study, perceived barriers ranged from structural (e.g., lack of financial resources and time) to psychological (e.g., embarrassment, fear of knowing the screening results). More educational materials should be provided to the community and clinics to help overcome these barriers to CRC screening. Future research should explore more effective interventional strategies to overcome these obstacles.

Apart from the factors identified in this study, the literature has suggested that cultural practices may play a role in cancer screening behaviour. Although government subsidies were available to support the cost of screening, 56.1% of the study participants still remained unscreened. The feedback that we collected from the participants suggested that most Chinese individuals do not see a need for screening in the absence of symptoms [31, 32]. They do not give preventive healthcare a high priority, and they prefer to spend their time doing household chores or working. Even when there is a problem, when compared to other ethnic groups such as Hispanics or African Americans, Chinese prefer to first seek Eastern forms of health management before seeking Western medical care [33]. Furthermore, in Chinese culture, cancer is considered unpreventable, and Chinese people believe they are at a lower risk of developing cancer than Westerners [34]. As a result, Chinese people who hold these cultural views tend to avoid colorectal cancer screening.

### Limitations

This study has some limitations. One limitation was the sampling method. The participants were recruited via convenience sampling in the community. We approached the participants mainly during office hours or when activities were held in the centres. These participants were generally active members of the community. Thus, we may have missed non-active community members or those who do not use centre services. Selection bias may therefore be a problem, and this may limit the generalisability of the study findings to the rest of the population in the territory. Additionally, screening uptake was self-reported, and participants may have had a tendency to provide socially desirable answers. In future studies, it is suggested that screening attendance be reviewed potentially using medical records. Finally, in view of the cross-sectional nature of the study, no causal relationships can be guaranteed for the identified screening uptake-associated factors.

## Conclusion

The CRC screening uptake rate in Hong Kong has increased over recent decades, although it remains lower than that in other Asian or Western countries. We found that CRC screening uptake among Chinese individuals aged 50 to 75 years was affected by various predisposing, enabling, and need-for-care factors. Among all participants, the provision of a government subsidy for screening was the most important enabling factor for FOBT/FIT uptake. Perceived barriers to screening remain an important predisposing factor that lower participants’ uptake of screening. Continual efforts are warranted to promote the subsidised screening programme, and relevant educational materials that address the barriers identified in this study should be developed and disseminated in the community.

## Data Availability

The datasets used and/or analysed during the current study are available from the corresponding author on reasonable request.
